# A Transcriptomic Regulatory Network among miRNAs, lncRNAs, circRNAs, and mRNAs Associated with L-leucine-induced Proliferation of Equine Satellite Cells

**DOI:** 10.3390/ani13020208

**Published:** 2023-01-06

**Authors:** Jingya Xing, Xingzhen Qi, Guiqin Liu, Xinyu Li, Xing Gao, Gerelchimeg Bou, Dongyi Bai, Yiping Zhao, Ming Du, Manglai Dugarjaviin, Xinzhuang Zhang

**Affiliations:** 1 Inner Mongolia Key Laboratory of Equine Genetics, Breeding and Reproduction, Scientific Observing and Experimental Station of Equine Genetics, Breeding and Reproduction, Ministry of Agriculture and Rural Affairs, Equine Research Center, College of Animal Science, Inner Mongolia Agricultural University, Hohhot 010018, China; 2 Liaocheng Research Institute of Donkey High-Breeding and Ecological Feeding, College of Agronomy, Liaocheng University, Liaocheng 252000, China

**Keywords:** L-leucine, equine, satellite cells, RNA-seq, non-coding RNA, ceRNA network

## Abstract

**Simple Summary:**

The Mongolian horse, one of the unique breeds in China, has excellent endurance quality formed by the half-grazing and half-wild breeding feeding mode on the grassland. However, the muscle of horses is prone to mechanical damage in the process of exercise, which can reduce the performance of horses and even cause a series of diseases. Mammalian skeletal muscle satellite cells (SCs) are located between the sarcolemma and the basal lamina of the muscle fiber. The SCs will be activated once the muscles are injured. L-leucine, as an important essential amino acid, plays an important role in the synthesis and metabolism of muscle proteins and the composition of muscle fibers. Therefore, the study on the mechanism of L-leucine regulating the proliferation of equine SCs can not only improve the exercise performance of horses but also reduce muscle injury of horses during exercises. The results of this study reveal the mechanism of L-leucine promoting the proliferation of equine skeletal muscle satellite cells.

**Abstract:**

In response to muscle injury, muscle stem cells are stimulated by environmental signals to integrate into damaged tissue to mediate regeneration. L-leucine (L-leu), a branched-chain amino acid (BCAA) that belongs to the essential amino acids (AAs) of the animal, has gained global interest on account of its muscle-building and regenerating effects. The present study was designed to investigate the impact of L-leu exposure to promote the proliferation of equine skeletal muscle satellite cells (SCs) on the regulation of RNA networks, including mRNA, long non-coding RNA (lncRNA), covalently closed circular RNA (circRNA), and microRNA (miRNA) in skeletal muscles. Equine SCs were used as a cell model and cultured in different concentrations of L-leu medium. The cell proliferation assay found that the optimal concentration of L-leu was 2 mM, so we selected cells cultured with L-leu concentrations of 0 mM and 2 mM for whole-transcriptiome sequencing, respectively. By high-throughput sequencing analysis, 2470 differentially expressed mRNAs (dif-mRNAs), 363 differentially expressed lncRNAs (dif-lncRNAs), 634 differentially expressed circRNAs (dif-circRNAs), and 49 differentially expressed miRNAs (dif-miRNAs) were significantly altered in equine SCs treated with L-leu. To identify the function of autoimmunity and anti-inflammatory responses after L-leu exposure, enrichment analysis was conducted on those differentially expressed genes (DEGs) related to lncRNA, circRNA, and miRNA. The hub genes were selected from PPI Network, including ACACB, HMGCR, IDI1, HAO1, SHMT2, PSPH, PSAT1, ASS1, PHGDH, MTHFD2, and DPYD, and were further identified as candidate biomarkers to regulate the L-leu-induced proliferation of equine SCs. The up-regulated novel 699_star, down-regulated novel 170_star, and novel 360_mature were significantly involved in the competing endogenous RNA (ceRNA) complex network. The hub genes involved in cell metabolism and dif-miRNAs may play fundamental roles in the L-leu-induced proliferation of equine SCs. Our findings suggested that the potential network regulation of miRNAs, circ-RNAs, lncRNAs, and mRNAs plays an important role in the proliferation of equine SCs, so as to build up new perspectives on improving equine performance and treatment strategies for the muscle injuries of horses.

## 1. Introduction

The dietary supplementation of AAs over those required for protein synthesis may have adverse effects, such as metabolic disturbances and reduced cell proliferation, but could be beneficial in certain situations. Therefore, the appropriate concentration of AAs is conducive to body development and cell growth [1]. Meanwhile, it is also a potent regulator of protein synthesis in healthy skeletal muscle and muscular atrophy [2,3,4,5]. It has been found that essential AAs stimulate the net balance of muscle proteins in healthy volunteers, and BCAAs promote protein synthesis to alleviate muscle pathologies in DMD mice [6,7,8]. Leucine (Leu), isoleucine, and valine belong to the BCAA, which makes up about one-third of muscle protein. Leucine has the highest oxidation rate among BCAA. Leucine has been used as a dietary supplement for athletes and older humans. [9,10]. Particularly, Leu has been proven to accelerate the synthesis process of protein in rats at a very early age [11] and has been proposed as a “pharmaceutical nutrient” to prevent muscle atrophy or repair muscle damage in older adults [12]. In early studies, the potential abilities of Leu to improve human muscle protein synthesis and health conditions had been proved, but the efficacious dose of Leu supplement is still unknown [12,13]. Nowadays, horses are mainly used for competitive racing and begin rigorous training at the age of one and a half years [14]. Maintaining speed, endurance, physical fitness, and desirable behavioral characteristics are critical in equine racing [15]. Horses are prone to suffering muscle injuries during intense exercise. In response to muscle injury, SCs integrate environmental signals in the damaged tissue to mediate regeneration. Although SCs are mitotically quiescent, they activate in response to injury; then, they re-enter the cell cycle and begin to proliferate, differentiate, and fuse, eventually regenerating myofibers [16]. These environmental signals are tightly regulated to ensure the expansion of the SC population to repair the damaged myofibers while allowing the re-proliferation of SCs [17,18]. Therefore, it is a crucial milestone in the equine racing career to improve performance and repair the damage after exercise. Now, racing jurisdictions have enacted regulations based on the rigorous practices of most thoroughbred racing countries that prohibit the presence of all compounds that may affect the body systems [19]. In previous papers, AAs had extensive application in food supplements, as well as activating mTORC1 to accumulate muscle synthesis. [20,21,22,23]. In particular, Leu intake is not only associated with increased protein accretion and muscle regeneration but also participates in the anabolic signaling pathway in skeletal muscles, although the overall effect is limited [12,24,25]. Previous studies also demonstrated that Leu treatment significantly altered the cellular amino acid metabolism and the fatty acid β-oxidation pathway in human cells, respectively [26,27]; these results implied that Leu could play other roles. Thus, we are interested in further investigating the mechanisms that may account for the L-leu-induced proliferation of equine SCs.

Discoveries in the past decade have identified that the contributions of RNA inside mammalian cells are much more than messenger functions [28]. Each discovery unveiled a new layer of mystery in relation to biological regulation and function. Meanwhile, as a research hotspot, research into non-coding RNAs (ncRNAs) started in the 1950s. [29]. RNA species beyond mRNA include intronic RNAs, microRNAs (miRNAs), long non-coding RNAs (lncRNAs), circular RNAs (circRNAs), and extracellular RNAs [30]. Increasingly, further research shows that the function of ncRNAs in biological systems cannot be ignored. Although ncRNAs were unable to function as messengers to encode proteins, they can affect the expression of other genes through alternate mechanisms. [31,32,33]. There is evidence that ncRNAs are involved in the relationship between mouse myogenesis and striated muscles. [34]. Recently, some miRNAs have been found to regulate the proliferation and differentiation of rabbit and bovine SCs, which is another advance in the study of skeletal muscle development at the molecular level [35,36]. The extensive expression of ncRNAs in muscle has been shown to play a critical regulatory role [37]. NcRNAs will contribute to broadening our understanding of the controlling factors of skeletal muscle function, muscle regeneration, and disorders. These findings have important implications for improving equine performance and repairing muscle damage.

The object of this study is to establish a network of lncRNAs, mRNAs, circRNAs, and miRNAs in SCs in response to an appropriate concentration of L-leu exposure. We provide a mechanistic explanation for the proliferation of SCs associated with L-leu.

## 2. Materials and Methods

### 2.1. Materials

The following materials are used in the cell culture: L-leu (#L8912), lysine (Lys, #L9037), arginine (Arg, #A6969), Collagen Ⅰ (#SCR103), Basic Fibroblast Growth Factor (bFGF, #13256-029), BSA (A1933), and DMSO (#C6295), which were purchased from Sigma-Aldrich, USA. DMEM (#11995065), L-leu-deprived DMEM powder (#88425), fetal bovine serum (FBS, #10099141C), Antibiotics-antimycotic (#15240062), and Dulbecco phosphate-buffered saline (DPBS, #14190144) were purchased from Gibco, Life, Technologies, Grand Island, NY, USA. Culture dish (10 and 6 cm), Cell ware 6, 24, and 96-well plates, Matrigel and cell strainer, were purchased from Corning, NY, USA. Accutase, Cell Detachment Solution was purchased from innovative cell technologies, SD, USA. Mouse monoclonal anti-PAX7 (#sc-81648) was purchased from Santa Cruz Biotechnology, CA, USA. Donkey anti-Mouse IgG (H + L) was purchased from Invitrogen (#A10036, Invitrogen, Carlsbad, CA, USA). DAPI (#C0065), Triton X-100 (#9002-93-1), and Tween 20 (#9008-64-5) were purchased from Solarbio (Beijing, China). CCK 8 was purchased from Good Laboratory Practice Bioscience, Montclair, CA, USA. 

### 2.2. Animals and Sample Collection

Semitendinosus muscle samples were aseptically collected from a 6-month-old healthy colt in a commercial abattoir during routine slaughter. The slaughter procedures and all of the sample collections specifically complied with the guidelines approved by the Animal Welfare Committee of Inner Mongolia Agricultural University in relation to the experimental animals. Briefly, approximately 5 g of semitendinosus muscle was removed and soaked in cold DPBS containing 1% antibiotics. Finally, the tissue was taken back to the laboratory for the extraction of equine primary SCs.

### 2.3. Primary Culture of Equine Satellite Cells

The semitendinosus muscle sample was washed with DPBS, and the visible connective tissue was cut out, washed again, and minced with scissors in 0.2% type Ⅰ collagenase solution. The sample was placed on a shaking table and digested in a 37 ℃ biochemical incubator for 1 h. Then, the cells were centrifuged at 1500× *g* for 5 min, and the supernatant was removed. Subsequently, they were sifted through 100-μm and 70-μm cell strainers, respectively, and centrifuged again at 1500× *g* for 5 min, and the supernatant was discarded again. Finally, the pellet was suspended in the proliferation medium supplemented with DMEM (11995065), 10% FBS, 1% Antibiotics-antimycotic, and 2.5 ng/mL bFGF. The differential adhesion technique was used to purify the equine SCs in order to decrease fibroblast contamination. Briefly, the suspended cells were placed on matrigel-coated dishes for two hours; then, the supernatant was transferred to a new matrigel-coated dish. After a proliferation medium was added, the samples were placed in 5% CO_2_ at 37 ℃, and the growth medium was changed after two days. Then, the purification process was repeated so that the purity of the equine SCs was beyond 90%. On the 7th day of proliferation, the cells were digested with accutase at 90% confluence and suspended in FBS with 10% DMSO. The cells gradually cooled down to −80 ℃ and were then frozen in liquid nitrogen for subsequent use.

### 2.4. Immunofluorescence

The cells were stained with PAX7 antibodies to determine the purity of the SCs. The cells were cultured in a 24-well matrigel-coated plate (three wells of cells per replication) and were fixed in 4% paraformaldehyde for 15 min at room temperature; then they were infiltrated with DPBS containing 0.1% Triton X-100 for 30 min. After being sealed with 1% BSA solution for 1 h, the 24-well plate was placed in a refrigerator at 4 ℃ and incubated overnight with the diluted primary antibody of PAX7 (1:200). After being washed with a cleaning solution (DPBS + 0.1% Tween 20 + 0.1% Triton X-100) three times, donkey anti-mouse IgG secondary antibody and Alexa Fluor 546 conjugate (1:500) were added, and then incubated in a room temperature shaker for 1 h, for the detection of PAX7 in the nucleus (color: red). Then, they were washed with cleaning solution three times, and 200 μL of DAPI solution was added and incubated for 15 min at room temperature. Then it was washed once with a cleaning solution, and, finally, an anti-fluorescence quenching agent was added, and it was shaken well on the shaking table. The images were taken using an inverted fluorescence microscope within 30 min. 

### 2.5. Cell Proliferation Rate Assay

For the L-leu-deprived DMEM, the DMEM powder (without L-leu, Arg and Lys, 88425) was supplemented at 84 mg/L and 146 mg/L (final concentration), and the pH value was adjusted to be consistent with DMEM. The third-generation cells were accurately detached and counted using a CellDrop Cell Counter (Denovix, Wilmington, DE, USA) after reaching approximately 90% confluence. The same number of cells (4000) were transferred to a 96-well plate overnight culture, later replaced with the L-leu-deprived DMEM for 12 h. Following starvation, we cultured the cells in an L-leu-deprived medium supplemented with various concentrations of L-leu (0 mM, 0.5 mM, 1 mM, 2 mM, 5 mM, and 10 mM) for 24 h, 48 h, and 72 h, respectively (three wells of cells for per concentration). We changed the medium after two days during the experiment. Subsequently, 10 μL of CCK-8 solution was added into each well and then incubated for another 4 h. The optical density (OD) value was measured at 450 nm using a microplate reader.

### 2.6. Experimental Design

As described above, the third-generation cells were cultured and counted, and the same number of cells (40,000) was transferred to the ten matrigel-coated culture dishes (6 cm) overnight. Firstly, all of the cells were starved in L-leu-deprived DMEM for 12 h. Then, ten dishes of SCs were randomly divided into the control group (CON) and L-leu supplemented group (LEU) and were cultured in the L-leu-deprived medium and the 2 mM (final concentration) supplemented medium, respectively. All of the cells underwent the process for 48 h at 37 ℃ under 5% CO_2_. After processing, the medium from each plate was discarded, and the cells were collected. Finally, they were stored at −80 ℃ until further analysis. For each group, five biological replicates were performed for each group to increase the statistical confidence.

### 2.7. RNA Extraction, Library Preparation and High-through Sequencing

The total RNA was extracted using the mirVana miRNA Isolation Kit (Ambion-1561, Thermo Fisher, Waltham, MA, USA) according to the manufacturer’s protocol. The integrity of the RNA was evaluated using the Agilent 2100 Bioanalyzer (Agilent Technologies, Santa Clara, CA, USA). The sequencing libraries were constructed by removing the ribosomal RNA with the Ribo-Zero Gold rRNA Removal Kit (Illumina, RS-122-2301, Beijing, China) according to the manufacturer’s manual. After the rRNA-depleted RNA was fragmented, the sequencing library was constructed. Briefly, the first-strand cDNA was synthesized using 8 μL of First Strand Synthesis Act D Mix and SuperScript II Reverse Transcriptase (Invitrogen, Carlsbad, CA, USA). Then, 5 μL End Repair Control and 20 μL Second Strand Marking Master Mix were adopted for the synthesis of the second strand cDNA. The double strands of cDNA were purified by using AMPure XP beads (BECKMAN COULTER, Brea, CA, USA). After adenylate 3’ ends and adapters ligation, the DNA fragments were enriched through the PCR process. The PCR mixture was incubated in the following progress: 1 cycle at 98 ℃ for 30 s; 15 cycles at 98 ℃ for 10 s, 60 ℃ for 30 s, 72 ℃ for 30 s; 1 cycle at 72 ℃ for 5 min; hold at 10 ℃. Finally, the products were purified by using Agencourt AMPure XP (BECKMAN COULTER, Brea, CA, USA), and the library quality was assessed on the Agilent Bioanalyzer 2100 system (Agilent Technologies, Santa Clara, CA, USA).

Following the ethanol-induced precipitation and centrifugal enrichment of the small RNA sample, the sequenced libraries extraction procedure was conducted according to the Illumina Small RNA sample Preparation Kit (RS-200-0012) purchased by Annoroad Gene Technology Corporation (Beijing, China).

### 2.8. Bioinformatics Analysis the Object of This Study Is to Establish a Network of lncRNAs, mRNAs, circRNAs, and miRNAs in SCs in Response to an Appropriate Concentration of Leu Exposuret

The raw reads generated during the high-throughput sequencing were fastq format sequences. To obtain high-quality reads that could be used for later analysis, the raw reads needed to be further quality filtered. The raw reads were filtered using the Trimmomatic (v 0.38, http://www.usadellab.org/cms/index.php?page=trimmomatic, accessed on 9 July 2022) [38] software to remove (1) the reads containing the sequencing adapter and primer sequence, (2) the reads that had a low-quality base (base quality less than 15), (3) the reads with N-bases (base quality less than 3), and (4) the reads with a length less than 50 bp. Using hisat2 (v 2.1.0, http://www.ccb.jhu.edu/software/hisat/, accessed on 9 July 2022) [39] the clean reads were aligned to the equine genome (NCBI_EquCab3.0). Subsequently, htseq-count (v 0.11.2, http://www-huber.embl.de/HTSeq, accessed on 9 July 2022) [40], Infernal (v 1.1.4, http://infernal.janelia.org, accessed on 9 July 2022) [41], and CIRI (v 2.0.3, https://sourceforge.net/projects/ciri/files/CIRI2/, accessed on 9 July 2022) [42] software were used separately to compare with the database to analyze the mRNA, lncRNA, and circRNA. Using eXpress (v 1.5.1, https://pachterlab.github.io/eXpress/, accessed on 9 July 2022) to analyze gene quantitative analysis, the FPKM value and counts value (the number of reads for each gene in each sample) were obtained. Differentially expressed lncRNAs and circRNAs were identified with DESeq (v 1.34.1, https://bioconductor.org/packages/release/bioc/html/DESeq2.html, accessed on 9 July 2022) [43] software, and the *p*-value < 0.05 and **|** log_2_ (fold change) **|** ≥ 2 were assigned as significantly dif-mRNAs, dif- lncRNAs, dif- circRNAs, and dif-miRNAs.

Then, the data were further processed and screened. The length distribution of the clean sequences in the reference genome was determined for the primary analysis. These RNAs were aligned and then subjected to the BLAST [44] search against Rfam (v 10.1) (http://www.sanger.ac.uk/software/Rfam, accessed on 9 July 2022) [45] and GenBank databases (v 253.0) (http://www.ncbi.nlm.nih.gov/genbank/, accessed on 9 July 2022). The known miRNAs were identified by aligning them against the miRNA database (v.22) (http://www.mirbase.org/, accessed on 9 July 2022) [46], and the known miRNA expression patterns in the different samples were analyzed. The unannotated small RNAs were analyzed using miranda (v.3.3a, http://www.microrna.org/microrna/home.do, accessed on 9 July 2022) [47] to predict the new miRNAs. Based on the hairpin structure of a pre-miRNA and the miRbase database, the corresponding miRNA star sequence was also identified. Identically differentially expressed miRNAs were identified with the thresholds of a *p*-value < 0.05 and | log_2_ (fold change) | ≥ 2.

### 2.9. Quantitative Real- Time PCR

Total RNA (from the CON group and LEU, *n* = 3) isolation was performed according to the protocol supplied with the miRNeasy Mini Kit (Qiagen, Hilden, Germany). The cDNA was synthesized using PrimeScript^TM^ RT Master Mix (TaKaRa, Dalian, China) according to the manufacturer’s instructions. The RNA quantity and cDNA were measured using an Epoch Microplate Spectrophotometer (Agilent Technologies, Santa Clara, CA, USA) and agarose. The gene-specific primers used are listed in Supplementary Table S1, and the primers were synthesized by Sangon Biotech (Beijing, China). The gene expression was determined using the TB Green^®^ Premix Ex Taq™ II (TaKaRa, Dalian, China) according to the manufacturer’s instructions on a CFX96 PCR instrument (Biored, Philadelphia, PA, USA). Finally, the relative gene expression was determined using the comparative 2^−△△CT^ method, and GAPDH was used as an endogenous control.

### 2.10. Statistical analysis

The quantitative data were analyzed through variance using the GLM program in SAS (v 8.0) software, and multiple comparisons were made using Duncan’s method. We considered a *p*-value < 0.05 to indicate significant differences between the groups.

### 2.11. GO and KEGG Enrichment Analysis of the dif-mRNA, dif-lncRNA, dif-circRNA and dif-miRNA

Selecting differential transcripts with a *p*-value < 0.05 and | log_2_ (fold change) | ≥ 2, and analyzing differential mRNA GO (http://www.gpmaw.com/html/swiss-prot.html, accessed on 9 July 2022) and KEGG (http://www.genome.jp/tools/kaas/, accessed on 9 July 2022) enrichment were performed with the Hypergeometric Distribution Test. The *p*-value and *q*-value were used to test the reliability of the analysis.

### 2.12. PPI Network and Module Analysis of dif-mRNA

The STRING (v 10.0; https://string-db.org/cgi/input.pl, accessed on 20 July 2022) database was utilized for the analysis of the interactions between the DEG-encoded proteins [48]. All of the DEGs were set by Cytoscape software (v 3.2.0; https// cytoscape.org/, accessed on 20 July 2022). The CytoNCA [49] plug-in (v 2.1.6, https://apps.cytoscape.org/apps/cytonca, accessed on 20 July 2022) was used to analyze the topological network properties of the node. The important nodes of the PPI network can be determined by ranking the score of every node. The most remarkable clustering modules in the PPI network were processed by the MCODE (v 1.4.2, https://apps.cytoscape.org/apps/MCODE, accessed on 20 July 2022) [50] plug-in of the Cytoscape. A score of ≥5 was considered the threshold. The GO enrichment analysis was carried out for the significant clustering module genes.

### 2.13. Prediction of miRNA Regulation Relationship

The targets of the differentially expressed miRNAs were predicted using Miranda software (v 3.3a) [51] in animals with the following parameters: S ≥ 150 ΔG ≤ −30 kcal/mol and require strict 5’ seed pairing. We combined the predicted miRNA-gene relationship together with the dif-mRNA to acquire the dif-miRNA-dif-mRNA regulatory relationship. Miranda was used to predict the regulatory relationships of miRNA-lncRNA and miRNA-circRNA.

### 2.14. Co-Expression Analysis of dif-lncRNA-dif-mRNA and dif-circRNA-dif-mRNA

The matrix data of dif-mRNA, dif-lncRNA, as well as dif-circRNA were calculated to generate the correlation coefficients. The correlation tests were conducted to screen the significant dif-mRNA-dif-lncRNA and dif-mRNA-dif-circRNA to obtain the co-expression relationship between dif-mRNA-dif-lncRNA and dif-mRNA-dif-circRNA.

### 2.15. Competing Endogenous RNA (ceRNA) Network Analysis

It is well known that miRNAs are a class of endogenous ncRNAs with regulatory functions, and their size is about 20–25 nucleotides long. A succession of nucleases cut mature miRNAs, which are assembled to attach to mRNA and silence genes. The purpose is to direct the degradation of the targets of silencing complexes or to block the translation of mRNAs. We constructed a ceRNA regulatory network by integrating the expression profiles and the regulation of the mRNAs, lncRNAs, circRNAs, and miRNAs. Further, we set up the lncRNA-mRNA co-expression relationship and the regulatory relationship between miRNA and lncRNA-mRNA. According to the results, lncRNA-mRNA was found to be only positively correlated. Therefore, we were more concerned with the miRNAs that can regulate both lncRNA-mRNA and miRNAs that could regulate the positive correlation co-expression relationship between mRNA and lncRNA were further obtained. The ceRNA regulatory network was formed through Cytoscape.

Similarly, we have integrated the circRNA-mRNA co-expression relationship and further obtained the regulatory relationship between miRNA, circRNA, and mRNA, respectively. Consistent with the above results, the circRNA-mRNA only has positive correlations. Furthermore, the positive correlation and co-expression relationship between mRNA and circRNA regulated by these miRNAs were further analyzed. A circRNA-miRNA-mRNA network was constructed by Cytoscape.

Integrating the two complex networks of lncRNA-miRNA-mRNA and circRNA-miRNA-mRNA, we focused on screening miRNAs that can regulate lncRNA, circRNA, and mRNA. Thus, we could achieve a positive correlation between these miRNAs regulating the expression of these three RNAs.

## 3. Results

### 3.1. Culture and Identification of Equine Satellite Cells

**Figure 1:**
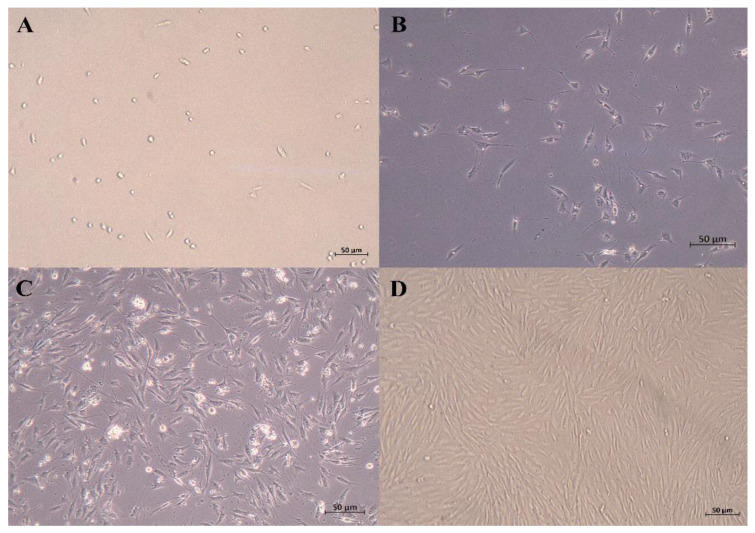
Micrographs of primary equine SCs. (**A**) just attached to the wall; (**B**) 24 h after isolation; (**C**) 48 h after isolation (typical SCs, with high refractive index and spindle shape); (**D**) 72 h after isolation (50 μm).

After being cultured for 24 h, some SCs began to adhere to the wall. The cells that were just attached to the wall were round, and some of the cells gradually extended, showing a short spindle shape and high refractivity index. Skeletal muscle satellite cells were fully adherent and were extended at 48 h. As shown in Figure 1, the SCs proliferated rapidly and were arranged in parallel when cultured for 72 h.

**Figure 2:**
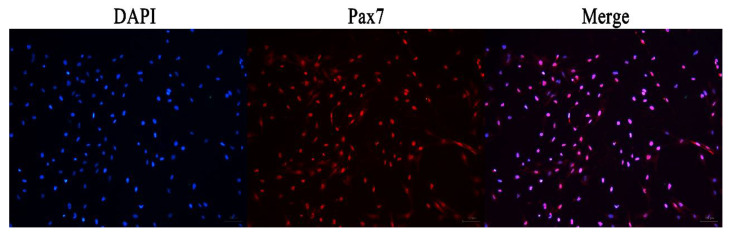
Immunofluorescence detection the expression of PAX7 protein in primary equine SCs.

**Figure 3:**
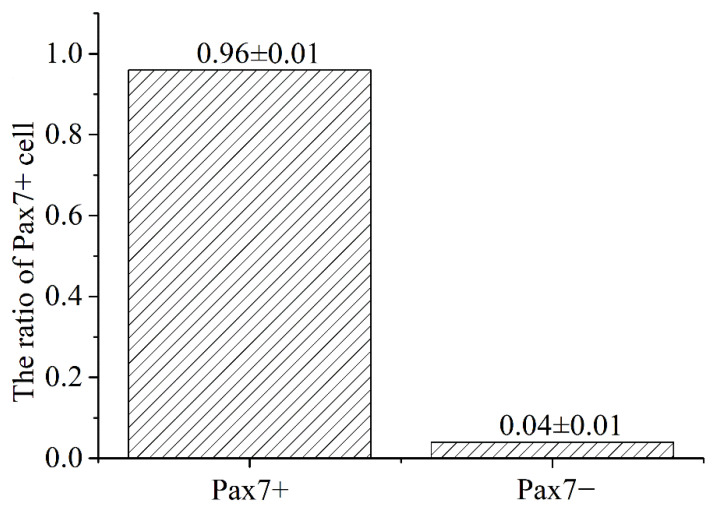
Statistics analysis on the purity ratio of PAX7+ using immunofluorescence staining. Result was presented as mean ± SEM.

The immunofluorescence analysis showed that the nuclei of the primary equine SCs were stained with red, indicating that PAX7 was expressed in the nucleus of the cells. The results showed that the fluorescence activity of PAX7 reached more than 96%; that is, the purity of primary equine SCs reached more than 96% (Figure 2 and Figure 3).

### 3.2. L-leucine Promotes Primary Satellite Cells Proliferation

**Figure 4:**
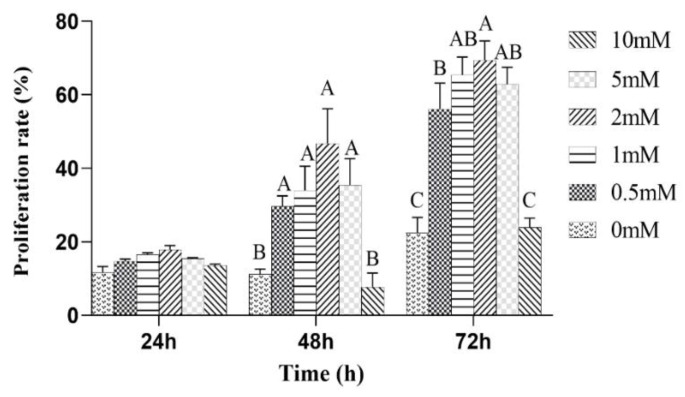
The effect of L-leu on the activity of equine SCs, and with different capital letter superscripts mean significant difference (*p* < 0.01), while with the same or letter superscripts mean no significant difference (*p* > 0.05).

The effects of the different concentrations of L-leu on the proliferation of equine SCs were observed. The results showed that L-leu enhanced the proliferation of equine SCs in a dose-dependent manner through the CCK-8 assays. There was no significant difference among all of the groups after a 24 h cell culture (*p* > 0.05). Then, 0 mM and 10 mM were significantly lower than other groups at 48 h and 72 h (*p* < 0.01), and 2 mM was significantly higher than the 0.5 mM group at 72 h (*p* < 0.01). Therefore, 2 mM was the optimal supplemental level after treatments with different concentrations of L-leu (Figure 4).

### 3.3. Differential Expression Analysis

**Figure 5:**
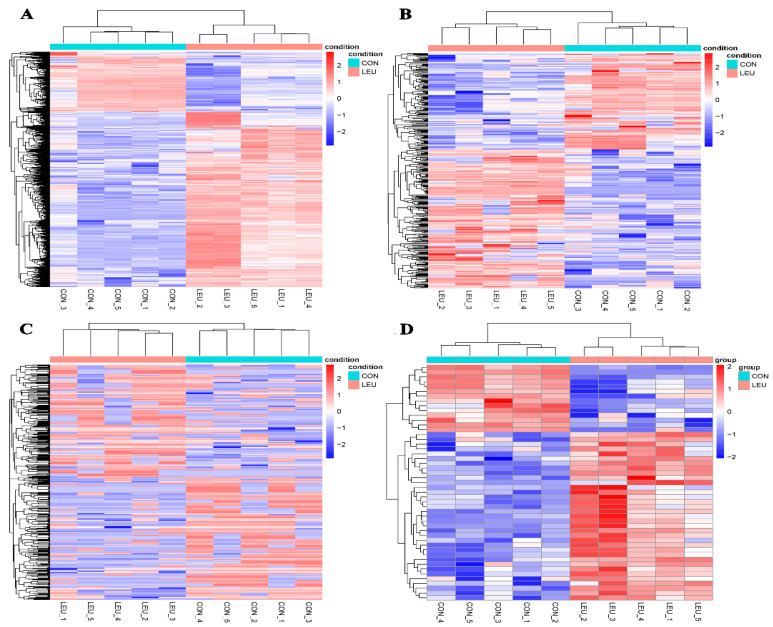
Heatmaps of Differentially Expression Molecules. (**A**) Heatmaps of differentially expressed mRNAs; (**B**) differentially expressed lncRNAs; (**C**) differentially expressed circRNAs; (**D**) differentially expressed miRNAs.

**Figure 6:**
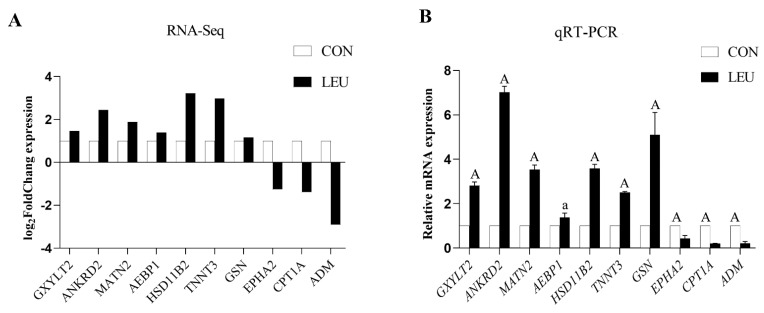
Histogram for RNA-Seq and qRT-PCR. (**A**) The histogram shows the expression level of selected genes detected by RNA-Seq. (**B**) The histogram shows the relative mRNA expression validated by qRT-PCR (*n* = 3 for each gene). X-axis shows the gene symbols, and Y-axis represents log_2_ (Fold Chang) expression and the relative mRNA expression, respectively. The values with different small letter superscripts mean significant difference (*p* < 0.05), and with a different capital letter superscripts mean extremely significant difference (*p* < 0.01).

The expression of the mRNA transcripts in the equine SCs in response to the L-leu treatment (2 mM) is represented by a Volcano Plot (Figure S1), and their *p*-value and fold changes are listed (Supplementary Table S2). The differentially expressed mRNA met the following rules: *p*-value < 0.05 and | log_2_ (fold change) | ≥ 2. According to the screening criteria, a total of 2470 dif-mRNAs were obtained, of which 1839 were up-regulated and 631 were down-regulated; 363 dif-lncRNAs were identified, of which 214 were up-regulated and 149 were down-regulated; a total of 634 dif-circRNAs were found, of which 309 were up-regulated and 325 were down-regulated; 49 dif-miRNAs were identified, of which 35 were up-regulated and 14 were down-regulated. The cluster diagram of dif-mRNAs, dif-lncRNAs, dif-circRNAs, and dif-miRNAs from the two groups are shown in Figure 5; we found that the LEU group can be significantly separated from the CON group, indicating that the results of the differential expression analysis showed high reliability. Ten dif-mRNAs were selected, and their expression levels were determined by qRT-PCR. All of the genes showed consistent results for both RNA-Seq and qRT-PCR (Figure 6). 

### 3.4. Functional Analyses of L-leucine Induced Differentially Expressed mRNAs

**Figure 7:**
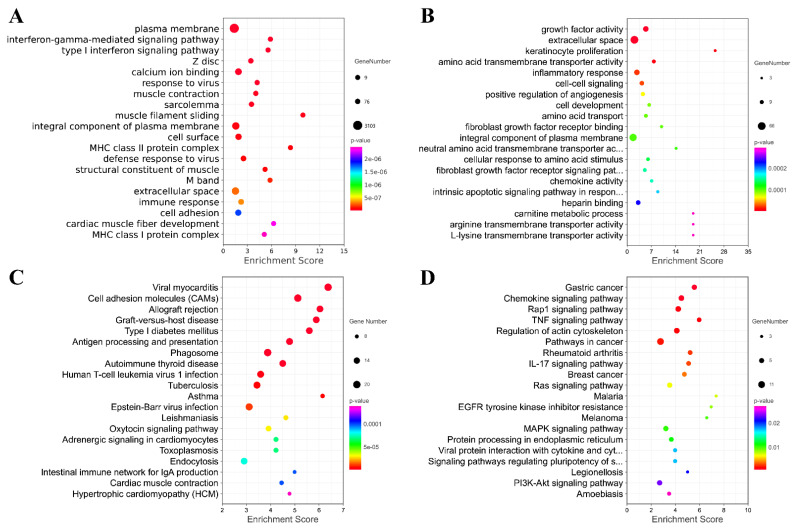
Analysis of GO and KEGG Pathway. Top 20 GO pathways enriched by up-regulated or down-regulated mRNA. (**A**) Top 20 GO pathways enriched by up-regulated mRNA; (**B**) Top 20 GO pathways enriched by down-regulated mRNA; (**C**) Top 20 KEGG pathways enriched by up-regulated mRNA; (**D**) Top 20 KEGG pathways enriched by down-regulated mRNA.

Furthermore, Gene Ontology (GO) and Kyoto Encyclopedia of Genes and Genomes (KEGG) were performed to reveal the potential function of the differentially expressed mRNAs (Figure 7). Figure 7 shows the top 20 GO and KEGG pathways enriched by the up-regulated or down-regulated dif-mRNA, respectively. As shown in the figure, the up-regulated genes were mainly enriched in the plasma membrane, interferon-gamma-mediated signaling pathway, type Ⅰ interferon signaling pathway, calcium ion binding, response to the virus, viral myocarditis, cell adhesion molecules (CAMs), and Antigen processing and presentation. Additionally, the down-regulated genes were significantly associated with growth factor activity, extracellular space, amino acid transmembrane transporter activity, inflammatory response, the chemokine signaling pathway and the rap1 signaling pathway, the TNF signaling pathway, and the regulation of actin cytoskeleton. We also found that the up-regulated and down-regulated genes were enriched into overlapping pathways, including the plasma membrane, an integral component of the plasma membrane, and extracellular space through GO analysis. In other words, their regulation methods are the opposite.

### 3.5. Protein–protein Interaction (PPI) Network

**Figure 8:**
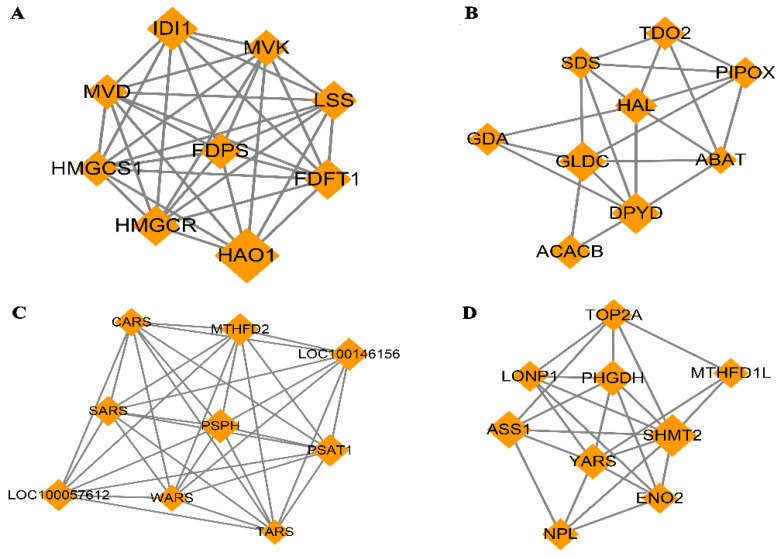
Four modules extracted from Protein–Protein Interaction (PPI) Network. Orange diamond represents degree value; large diamond has higher degree value. (**A**,**B**) Significant clustered up-regulated genes from the PPI; (**C**,**D**) Significant clustered down-regulated genes from the PPI.

The PPI network based on dif-mRNA consisted of 62 nodes and 181 interaction pairs. The nodes with high topological scores can be regarded as the key nodes in the network. Four modules were aggregated and extracted from the PPI networks using the MCODE (score ≥ 5) plug-in, of which the genes were up-regulated (Figure 8A,B) and down-regulated (Figure 8C,D). Module A (score = 8.75) contained nine nodes and 35 interaction pairs, including hydroxyacid oxidase (HAO1, degree = 29), isopentenyl-diphosphate delta isomerase 1 (IDI1, degree = 18), and 3-hydroxy-3-methyl glutarate monoacyl CoA reductase (HMGCR, degree = 16). Module B (score = 5.25) contained nine nodes and 21 interaction pairs, including dihydropymidine dehydrogenase (DPYD, degree = 19) and acetyl-coenzyme A carboxylase beta (ACACB, degree = 13). Module C (score = 8.75) contained nine nodes and 35 interaction pairs, including phosphoserine phosphatase (PSPH, degree = 31), loc100146156 (degree = 29), methylene terahydrofolate dehydrogenase (MTHFD2, degree = 28), and phosphoserine aminotransferase (PSAT1, degree = 28). Module D (score = 6) contained nine nodes and 24 interaction pairs, including serine hydroxy methyltransferase 2 (SHMT2, degree = 32), argininosuccinate synthetase (ASS1, degree = 25), and phosphoglycerate dehydrogenase (PHGDH, degree = 22).

**Figure 9:**
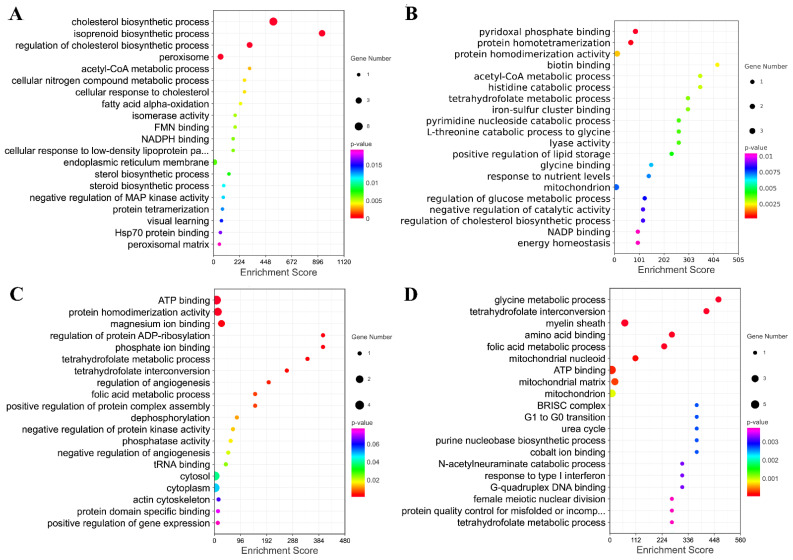
The GO Enrichment Analysis of Four Modules Extracted from PPI. (**A**–**D**) Top 20 GO terms enriched by genes in these four modules.

Furthermore, the genes in the key modules were used for the GO enrichment analysis. The top 20 terms for each module were selected for display (Figure 9A–D). The genes in module A were significantly involved in the regulation of cholesterol biosynthetic processes, isoprenoid biosynthetic processes, cholesterol biosynthetic processes, and peroxisome. The genes in module B were significantly associated with protein homotetramerization and pyridoxal phosphate binding. The down genes in module C were significantly associated with protein homodimerization activity, ATP binding, magnesium ion binding, and amino acid binding. The down genes in module D were significantly associated with folic metabolic processes, the myelin sheath, tetrahydrofolate interconversion, amino acid binding, and glycine metabolic processes. 

### 3.6. Enrichment Analysis of miRNA, lncRNA and circRNA-Related Target Genes

**Figure 10:**
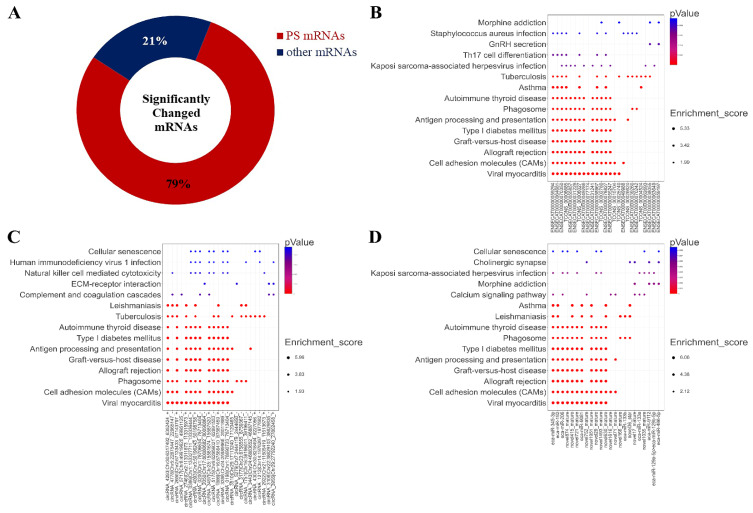
Significantly changed mRNAs after exposure to L-leu and bubble maps of KEGG Pathway Enrichment. (**A**) The ring indicated significantly changed mRNAs in the equine SCs after exposure to L-leu; (**B**–**D**) Gene enrichment pathways regulated by differentially expressed lncRNAs, cricRNAs, and miRNAs, respectively.

Surprisingly, we found that 1942 miRNAs were predicted as the targets of the altered miRNA after treatment with 2 mM L-leu for 48 h (Figure 10A); about 79% of the 2470 significantly changed mRNAs, the KEGG enrichment analysis was performed based on the mRNAs that are involved in the co-expression relationship between dif-lncRNA-dif-mRNA, dif-circRNA-dif-mRNA, and dif-miRNA-dif-mRNA. The results are shown in bubble diagrams (Figure 10B–D). The results showed that those target genes were significantly enriched in viral myocarditis, CAMs, allograft rejection, graft-versus-host disease, type Ⅰ diabetes mellitus, antigen processing, presentation and autoimmune thyroid disease, asthma, tuberculosis, leishmaniasis, human T-cell leukemia virus infection, and the intestinal immune network for IgA production.

### 3.7. ceRNA Network Construction

**Figure 11:**
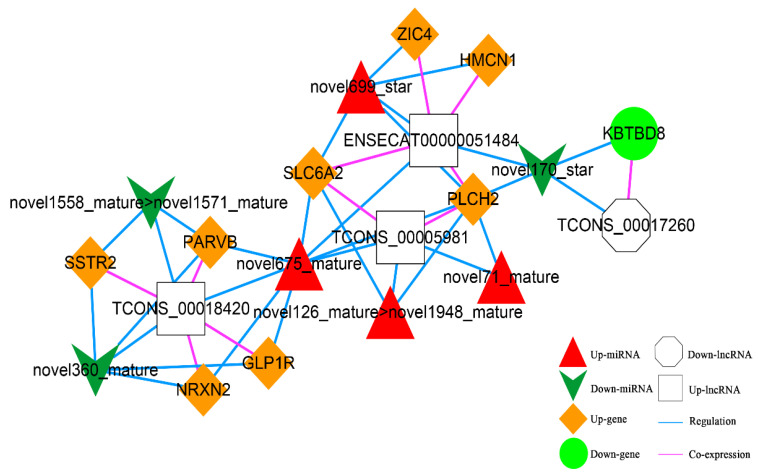
The lncRNA-miRNA-mRNA Network. Orange diamond and green ellipse represent the up-regulated and down-regulated genes, respectively; white rectangle and octagon show the up-regulated and down-regulated lncRNA, respectively; red triangle and green V indicate the up-regulated and down-regulated miRNA, respectively. The pink and blue line represents the co-expression and regulatory relationship, respectively.

According to the co-expression relationship of lncRNA-mRNA (top 100), combined with the regulatory relationship of dif-miRNA-dif-mRNA and dif-miRNA-dif-lncRNA, the lncRNA and mRNA were regulated by the same miRNA and the significantly differentially expressed were screened. In total, 41 lncRNA-miRNA-mRNA interactions were obtained (Figure 11), including three up-regulated and one down-regulated lncRNA, eight up-regulated and one down-regulated mRNAs, and four up-regulated and three down-regulated miRNAs.

**Figure 12:**
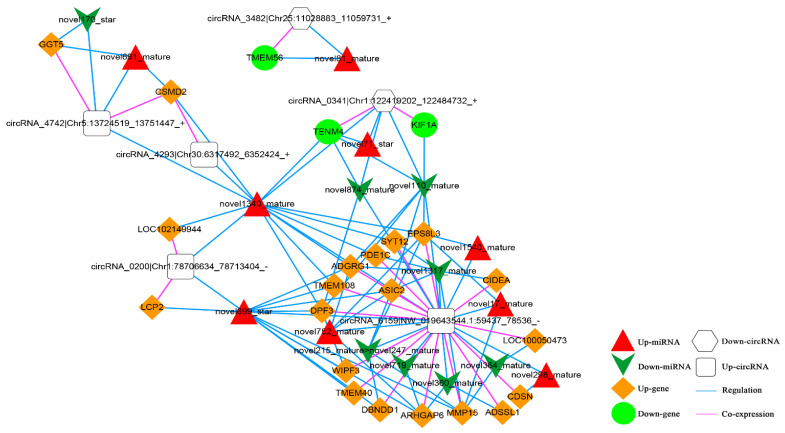
The circRNA-miRNA-mRNA Network. Orange diamond and green ellipse represent the up-regulated and down-regulated genes, respectively; white round rectangle and hexagon show the up-regulated and down-regulated circRNA, respectively; red triangle and green V indicate the up-regulated and down-regulated miRNA, respectively. The pink and blue line represents the co-expression and regulatory relationship, respectively.

Based on the co-expression relationship between circRNA and mRNA (top 100), as well as the regulatory link between dif-miRNA-dif-mRNA and dif-miRNA-dif-cirRNA, it was determined that the circRNA and mRNA were both significantly differentially expressed and controlled by the same miRNA and significantly differentially expressed were screened. The final results showed 105 circRNA-miRNA-mRNA interaction relationships. There are four up-regulated and two down-regulated circRNAs, 20 up-regulated and three down-regulated mRNAs, and nine up-regulated and eight down-regulated miRNAs. The circRNA-miRNA-mRNA network is shown in Figure 12.

**Figure 13:**
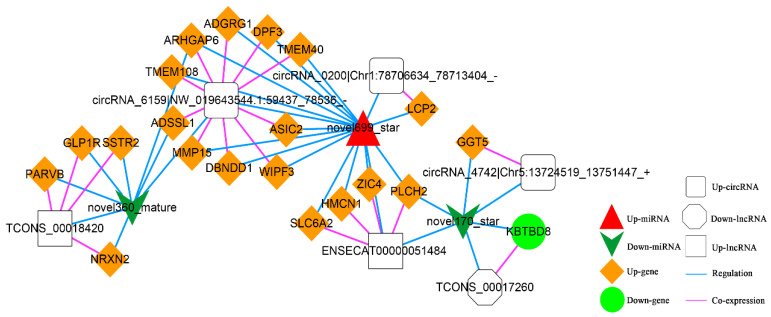
The Competing Endogenous RNA (ceRNA) Network. Orange diamond and green ellipse represent the up-regulated and down-regulated genes, respectively; white rectangle, round rectangle and octagon show the up-regulated lncRNA and circRNA, down-regulated lncRNA, respectively; red triangle and green V indicate the up-regulated and down-regulated miRNA, respectively. The pink and blue line represents the co-expression and regulatory relationship, respectively.

In addition, according to lncRNA-miRNA-mRNA and circRNA-miRNA-mRNA networks, differentially expressed circRNAs, lncRNAs, and mRNAs regulated by the same miRNA were further screened out. Finally, 52 interaction pairs were obtained (Figure 13), of which there were two up-regulated and one down-regulated lncRNA, three up-regulated circRNAs, 20 up-regulated and one down-regulated mRNA, and one up-regulated and two down-regulated miRNAs (novel699_star, up-regulated; novel170_star and novel360_mature, down-regulated).

## 4. Discussion

In young mammals and adults, SCs are stimulated by environmental factors in the tissue to mediate continuous proliferation and differentiation and promote effective muscle development. Skeletal muscle regeneration is initiated by the proliferation and differentiation of SCs, which is essential for skeletal muscle growth, homeostasis, and post-injury repair [52,53]. The common methods used for primary SCs isolation are tissue block culture and enzymatic digestion to release cells from the sarcolemma and the basal lamina of the muscle fiber. [54]. We obtained primary equine SCs with a purity of 90–95% by digesting with type I collagenase, which were then purified using the Matrigel-coated method and pre-plating. Pax7 is a specific marker protein of SCs, which was identified using immunofluorescence technology [55]. This method is simple and can obtain high-purity equine SCs. Meanwhile, it will also provide a cell model for the study of the molecular regulation mechanism of equine skeletal muscle.

Leucine is one of the indispensable BCAAs and an essential regulator of muscle quality by controlling protein synthesis [56]. The results have demonstrated that Leu is not only crucial for protein synthesis in skeletal muscle tissue but is also involved in other functions in pigs, rats, and humans, such as the regulation of energy metabolism [57], immune function [58,59], and injury repair after exercise [60,61]. In our study, we examined the expression profiles of the miRNA, lncRNA, circRNA, and mRNA in equine SCs following exposure to Leu. In order to analyze these RNA species collectively, we established a potential network successfully. It has been established that L-leu has the capacity to trigger immunological response, protein synthesis, and other significant impacts in human muscles, which is compatible with our findings [60,62,63]. Several previous studies have shown that adequate Leu can increase the abundance and collagen remodeling of human SCs [61], promote porcine SCs differentiation [64], and participate in the regulation of the mTOR signaling pathway in chicken SCs [65]. Overall, increasing evidence suggests that Leu can promote cell growth, muscle development, and immune response.

Whole-transcriptome sequencing refers to the sum of all types of RNA that can be transcribed, including mRNA and ncRNA. Whole-transcriptome research on specific cells under specific conditions can simultaneously analyze four kinds of RNAs (mRNA, lncRNA, circRNA, and miRNA). The use of the same batch of samples not only improves the comparability of study results but also enables ceRNA network regulation analysis of more than two RNA types. Whole-transcriptome sequencing reduced the screening scope and excavated the key genes. However, other researchers found that high false positives of whole-transcriptome sequencing could mislead the results when the coverage level is high [66]. In this study, we used high-throughput sequencing techniques to investigate the molecular pathways in equine SCs after exposure to L-leu. Mammalian skeletal muscle development is a very complex process, and the mechanism of SCs involved in repairing skeletal muscle regeneration has been studied for a long time. The destiny of SCs during the regeneration of skeletal muscle is determined by a complicated differentiation process that includes the following steps, differentiate and fuse with original skeletal muscle cells to form new muscle fibers [53]. The proliferation and differentiation of SCs during muscle regeneration are influenced by many factors, including nutrients, epigenetic regulation, and signal pathway-mediated regulators [17,18,67]. Following treatment with L-leu, dif-mRNAs clustered in pathways regulation to response to virus, CAMs, growth factor activity, amino acid transmembrane transporter activity, the chemokine signaling pathway, and the rap1 signaling pathway, which support the autoimmunity and anti-inflammatory response of the cell. Additionally, it may be a major fundamental reason for the L-leu-induced proliferation of equine SCs. Meanwhile, a few observations were particularly noteworthy; the functional enrichment analysis of differential mRNA, lncRNA, circRNA, and miRNA demonstrated that anti-inflammatory and cell adhesion were involved. Early research has found that CAMs are closely related to the immune system and can lead to immune recognition in human skeletal muscles [68]. Analysis of transcriptional pathways revealed that Leu was related to immunity and increased muscle function and older adults’ SCs abundance [61,69]. Inflammatory signaling can contribute to muscle atrophy and muscle damage and is associated with reduced strength in the elderly [70,71]. Importantly, inflammatory responses in human studies have been proven to lead to fibrosis accumulation, the impaired function of SCs, and contribute to poor skeletal muscle regeneration [72,73]. Moreover, Leu has been found to modulate protein turnover and immune response during human cancer cachexia [74]. Therefore, we speculate that inflammatory pathways may be reduced by L-leu in order to promote equine SCs proliferation (Figure 7). This also provides a theoretical basis for L-leu supplementation to treat post-exercise injuries in horses. Further investigation of this result mechanism is warranted.

As an essential BCAA, Leu supplementation not only replenished sufficient substrates for protein synthesis but also participated in cell metabolism to provide energy [75]. In this study, PPI network analysis identified the hub genes related to fat metabolism, amino acid metabolism, and nucleotide synthesis metabolism, including ACACB, HMGCR, IDI1, HAO1, SHMT2, PSPH, GLDC, PSAT1, ASS1, PHGDH, MTHFD2, and DPYD. Cell proliferation accompanied many changes in cellular metabolism to meet the biosynthetic demands. These hub genes were mainly involved in intracellular metabolic processes, which suggested that L-leu may improve cellular stress and anti-inflammatory response by regulating cellular metabolic processes. Current advances in cellular metabolism have shown that AA is not only a raw material for protein synthesis but also important regulators of cellular processes [76,77,78]. These metabolite genes may be related to the amino acid transfer pathway to skeletal muscle and the high activity of skeletal muscle tissue [79]. Activating transcription factor (ATF4) belongs to the basic Leu zipper (bZIP) transcription factor family, which has the consensus binding site cAMP responsive element (CRE). The abundance and deprivation of AA can lead to a series of reactions by the intracellular amino acid sensors [80], and then a large number of genes downstream of ATF4 can cope with AA starvation [81]. We found that the transcription level of ATF4 was down-regulated (Supplementary Table S2) with L-leu, which was significantly lower than the CON group. The results also showed that the genes downstream of ATF4 (PSPH, PSAT1, PHGDH and SHMT2) in the serine glycine synthesis pathway were down-regulated with L-leu treatment. The serine glycine synthesis pathway has a wider network that associates glycolysis with one-carbon metabolism and nucleotide synthesis, and now this has been the main object of study for inflammation and disease in animal cells [82]. L-leucine deficiency promotes the transcription level of ATF4 in order to balance the intracellular AAs. These findings indicate that L-leucine deficiency stimulates the serine–glycine biosynthesis pathway to provide additional glycine needed for increased protein synthesis is further in agreement with a recent report with AA deficiency in animal cells [80] and consistent with the result that pharmacologic inhibition of PHGDH and SHMT2 improve immune cell function in vivo in humans [82,83]. In other words, L-leu enhances the immune response in order to promote cell proliferation by inhibiting the serine and glycine biosynthesis pathways.

ASS1 is a key rate-limiting enzyme for the production of arginine, urea, and NO. The expression level of ASS1 in cells is also different due to the different metabolic pathways of Arg. ATF4 has been found to play an important role in the regulation of ASS1 [84]. Although there are few such studies, we speculate that ASS1 may also be a downstream transcription factor of ATF4, and its down-regulation is related to L-leu. This speculation requires further study. Moreover, mitochondrial folate enzyme (MTHFD2), as an important immunosuppressive and anticancer agent, is strongly up-regulated in human lymphocytes and cancers [85,86]. MTHFD2 is down-regulated with L-leu; it is a metabolic checkpoint that regulates cell fate and function. This may be caused by L-leu enhancing the higher pro-inflammatory cytokines of the whole host. L-leucine is a ketogenic amino acid, and its correlation with genes related to fat metabolism (ACACB, HMGCR, IDI1, and HAO1) and nucleotide metabolism (DPYD) is rarely reported. However, ACACB and HMGCR have been studied in metabolic syndrome, obesity, and tumors, and they have the function of promoting antitumor immunity. Currently, the research into Leu promoting muscle protein anabolism mainly focuses on humans and mice [74,87,88,89]. With the large size of the procedures involved in equine SCs proliferation, the genes that promote proliferation and muscle development remain to be discovered. These hub genes need to be investigated for future muscle development and muscle injury recovery in sports horses. The positive implications of L-leu supplementation indicate that it may reduce skeletal muscle loss, improve immune competence, and relieve muscle injury. Thus, L-leu may be a suitable nutritional strategy to hasten the healing of muscle damage brought on by exercise.

Additionally, we also found the critical role of up-regulated novel 699_star, down-regulated novel 170_star, and novel 360_mature were significantly enriched in the ceRNA complex network. These three miRNAs were newly predicted miRNAs in this experiment. It demonstrated that these miRNAs might play an essential role in the L-leu-induced proliferation of equine SCs. There are relatively few equine annotations in the miRbase database, which will also be supplementary data to the miRbase database. Future mechanistic studies are warranted to determine the role of these three miRNAs in the L-leu regulatory network of equine SCs.

## 5. Conclusions

In conclusion, this study explored the molecular mechanism of the L-leu-induced proliferation of equine SCs by using whole-transcriptome sequencing. The hub genes related to fat metabolism, amino acid metabolism, and nucleotide synthesis metabolism include ACACB, HMGCR, IDI1, HAO1, SHMT2, PSPH, PSAT1, ASS1, PHGDH, MTHFD2, and DPYD. They are involved in anti-inflammatory and cell adhesion processes and may play fundamental roles in the L-leu-induced proliferation of equine SCs. Novel 699_star, novel 170_star, and novel 360_mature may play a pivotal role in the mechanism of L-leu-promoting proliferation of equine SCs. These three miRNAs are newly predicted miRNAs, which will open a new sight in the study of L-leu improving exercise performance and post-exercise injury repair in horses. Meanwhile, these discoveries reveal the intricacy of the genome and offer possible new targets for L-leu-induced characterization. Excellent sports horses are very precious, especially thoroughbred horses. This is of great significance to solving the injury of horses after sports and improve their sports performance at home and abroad. To our knowledge, this is the first report where the global gene expression in L-leu-induced proliferation of equine SCs. It can provide a basis for subsequent animal experiments. However, this research is constrained by a cell model in proving a comprehensive overview of L-leu-induced proliferation of equine SCs, and horses belonging to the single stomach herbivore, compared with other animals, have unique physiological structures and eating habits. To establish the precise mechanism of occurrence, additional in vivo validation utilizing animal models and functional characterization are required.

## Data Availability

The datasets generated for this study can be found in NCBI SRA accession PRJNA915779 (https://www.ncbi.nlm.nih.gov/sra, accessed on 9 July 2022).

## Supplementary Materials

The following supporting information can be downloaded at: https://www.mdpi.com/article/10.3390/ani13020208/s1, Figure S1: Volcano plot of dif-mRNAs with RNA-Seq analyses in the Leu-induced proliferation of equine SCs; Table S1: Primers used for quantitative Real- Time PCR; Table S2: The *p*-value and fold changes of mRNA transcript expression in equine SCs with Leu (2 mM).

## References

[1] Cynober L. (2006). Introduction to the 5th amino acid assessment workshop. J. Nutr..

[2] Fujita S., Volpi E. (2006). Amino acids and muscle loss with aging. J. Nutr..

[3] Ham D.J., Murphy K.T., Chee A., Lynch G.S., Koopman R. (2014). Glycine administration attenuates skeletal muscle wasting in a mouse model of cancer cachexia. Clin. Nutr..

[4] Koopman R., Ly C.H., Ryall J.G. (2014). A metabolic link to skeletal muscle wasting and regeneration. Front. Physiol..

[5] Mok E., Violante C.E.-D., Daubrosse C., Gottrand F., Rigal O., Fontan J.-E., Cuisset J.-M., Guilhot J., Hankard R. (2006). Oral glutamine and amino acid supplementation inhibit whole-body protein degradation in children with Duchenne muscular dystrophy. Am. J. Clin. Nutr..

[6] Hulmi J.J., Lockwood C.M., Stout J.R. (2010). Effect of protein/essential amino acids and resistance training on skeletal muscle hypertrophy: A case for whey protein. Nutr. Metab..

[7] Davoodi J., Markert C.D., Voelker K., Hutson S., Grange R.W. (2012). Nutrition strategies to improve physical capabilities in Duchenne muscular dystrophy. Phys. Med. Rehabil. Clin..

[8] Lin C., Han G., Ning H., Song J., Ran N., Yi X., Seow Y., Yin H. (2020). Glycine enhances satellite cell proliferation, cell transplantation, and oligonucleotide efficacy in dystrophic muscle. Mol. Ther..

[9] Harper A., Miller R., Block K. (1984). Branched-chain amino acid metabolism. Annu. Rev. Nutr..

[10] Fukagawa N.K. (2013). Protein and amino acid supplementation in older humans. Amino Acids.

[11] Buse M.G., Reid S.S. (1975). Leucine. A possible regulator of protein turnover in muscle. J. Clin. Investig..

[12] Casperson S.L., Sheffield-Moore M., Hewlings S.J., Paddon-Jones D. (2012). Leucine supplementation chronically improves muscle protein synthesis in older adults consuming the RDA for protein. Clin. Nutr..

[13] Ohtani M., Kawada S., Seki T., Okamoto Y. (2012). Amino acid and vitamin supplementation improved health conditions in elderly participants. J. Clin. Biochem. Nutr..

[14] Ablondi M., Summer A., Vasini M., Simoni M., Sabbioni A. (2020). Genetic parameters estimation in an Italian horse native breed to support the conversion from agricultural uses to riding purposes. J. Anim. Breed. Genet..

[15] McGivney B., Hernandez B., Katz L., MacHugh D., McGovern S., Parnell A., Wiencko H., Hill E. (2019). A genomic prediction model for racecourse starts in the Thoroughbred horse. Anim. Genet..

[16] Sutcu H.H., Ricchetti M. (2018). Loss of heterogeneity, quiescence, and differentiation in muscle stem cells. Stem Cell Investig..

[17] Sakuma K., Yamaguchi A. (2018). Recent advances in pharmacological, hormonal, and nutritional intervention for sarcopenia. Pflügers Arch. Eur. J. Physiol..

[18] Consalvi S., Brancaccio A., Dall’Agnese A., Puri P.L., Palacios D. (2017). Praja1 E3 ubiquitin ligase promotes skeletal myogenesis through degradation of EZH2 upon p38α activation. Nat. Commun..

[19] Snow D.H. (1994). Ergogenic aids to performance in the race horse: Nutrients or drugs. J. Nutr..

[20] Frost R.A., Lang C.H. (2011). mTor signaling in skeletal muscle during sepsis and inflammation: Where does it all go wrong?. Physiology.

[21] Tan H.W.S., Sim A.Y.L., Long Y.C. (2017). Glutamine metabolism regulates autophagy-dependent mTORC1 reactivation during amino acid starvation. Nat. Commun..

[22] Saxton R.A., Sabatini D.M. (2017). mTOR signaling in growth, metabolism, and disease. Cell.

[23] Ham D.J., Caldow M.K., Lynch G.S., Koopman R. (2014). Arginine protects muscle cells from wasting in vitro in an mTORC1-dependent and NO-independent manner. Amino Acids.

[24] Bandt J. (2016). Leucine and Mammalian Target of Rapamycin–Dependent Activation of Muscle Protein Synthesis in Aging. J. Nutr..

[25] Magne H., Savary-Auzeloux I., Rémond D., Dardevet D. (2013). Nutritional strategies to counteract muscle atrophy caused by disuse and to improve recovery. Nutr. Res. Rev..

[26] Yan G., Li X., Peng Y., Long B., Fan Q., Wang Z., Shi M., Xie C., Zhao L., Yan X. (2017). The fatty acid β-oxidation pathway is activated by leucine deprivation in HepG2 cells: A comparative proteomics study. Sci. Rep..

[27] Zarfeshani A., Ngo S., Sheppard A.M. (2014). Leucine alters hepatic glucose/lipid homeostasis via the myostatin-AMP-activated protein kinase pathway-potential implications for nonalcoholic fatty liver disease. Clin. Epigenet..

[28] Morris K.V., Mattick J.S. (2014). The rise of regulatory RNA. Nat. Rev. Genet..

[29] Palazzo A.F., Lee E.S. (2015). Non-coding RNA: What is functional and what is junk?. Front. Genet..

[30] Matsui M., Corey D.R. (2017). Non-coding RNAs as drug targets. Nat. Rev. Drug Discov..

[31] Dai X., Zhang S., Zaleta-Rivera K. (2020). RNA: Interactions drive functionalities. Mol. Biol. Rep..

[32] Cech T.R., Steitz J.A. (2014). The noncoding RNA revolution—Trashing old rules to forge new ones. Cell.

[33] Kinoshita C., Aoyama K. (2021). The role of non-coding RNAs in the neuroprotective effects of glutathione. Int. J. Mol. Sci..

[34] Liu N., Williams A.H., Maxeiner J.M., Bezprozvannaya S., Olson E.N. (2012). microRNA-206 promotes skeletal muscle regeneration and delays progression of Duchenne muscular dystrophy in mice. J. Clin. Investig..

[35] Zhang W.R., Zhang H.N., Yi M.W., Yang D., Guo H. (2016). miR-143 regulates proliferation and differentiation of bovine skeletal muscle satellite cells by targeting IGFBP5. Vitr. Cell. Dev. Biol. Anim..

[36] Liu B., Yu S., He H., Cai M., Lai S. (2018). miR-221 modulates skeletal muscle satellite cells proliferation and differentiation. Vitr. Cell. Dev. Biol. Anim..

[37] Chen J.-F., Tao Y., Li J., Deng Z., Yan Z., Xiao X., Wang D.-Z. (2010). microRNA-1 and microRNA-206 regulate skeletal muscle satellite cell proliferation and differentiation by repressing Pax7. J. Cell Biol..

[38] Bolger A.M., Lohse M., Usadel B. (2014). Trimmomatic: A flexible trimmer for Illumina sequence data. Bioinformatics.

[39] Kim D., Langmead B., Salzberg S.L. (2015). HISAT: A fast spliced aligner with low memory requirements. Nat. Methods.

[40] Anders S., Pyl P.T., Huber W. (2015). HTSeq—A Python framework to work with high-throughput sequencing data. Bioinformatics.

[41] Nawrocki E.P., Eddy S.R. (2013). Infernal 1.1: 100-fold faster RNA homology searches. Bioinformatics.

[42] Gao Y., Wang J., Zhao F. (2015). CIRI: An efficient and unbiased algorithm for de novo circular RNA identification. Genome Biol..

[43] Anders S., Huber W. (2012). Differential Expression of RNA-Seq Data at the Gene Level—The DESeq Package.

[44] Altschul S.F., Gish W., Miller W., Myers E.W., Lipman D.J. (1990). Basic local alignment search tool. J. Mol. Biol..

[45] Griffiths-Jones S., Bateman A., Marshall M., Khanna A., Eddy S.R. (2003). Rfam: An RNA family database. Nucleic Acids Res..

[46] Griffiths-Jones S., Saini H.K., Van Dongen S., Enright A.J. (2007). miRBase: Tools for microRNA genomics. Nucleic Acids Res..

[47] Friedländer M.R., Mackowiak S.D., Li N., Chen W., Rajewsky N. (2012). miRDeep2 accurately identifies known and hundreds of novel microRNA genes in seven animal clades. Nucleic Acids Res..

[48] Zhang Y.J., Ma Y.S., Xia Q., Yu F., Lv Z.W., Jia C.Y., Jiang X.X., Zhang L., Shao Y.C., Xie W.T. (2018). MicroRNA-mRNA integrated analysis based on a case of well-differentiated thyroid cancer with both metastasis and metastatic recurrence. Oncol. Rep..

[49] Tang Y., Li M., Wang J., Pan Y., Wu F.-X. (2015). CytoNCA: A cytoscape plugin for centrality analysis and evaluation of protein interaction networks. Biosystems.

[50] Bandettini W.P., Kellman P., Mancini C., Booker O.J., Vasu S., Leung S.W., Wilson J.R., Shanbhag S.M., Chen M.Y., Arai A.E. (2012). MultiContrast Delayed Enhancement (MCODE) improves detection of subendocardial myocardial infarction by late gadolinium enhancement cardiovascular magnetic resonance: A clinical validation study. J. Cardiovasc. Magn. Reson..

[51] Enright A., John B., Gaul U., Tuschl T., Sander C. (2003). MicroRNA targets in Drosophila. Genome Biol..

[52] Moss F.P., Leblond C.P. (1971). Satellite cells as the source of nuclei in muscles of growing rats. Anat. Rec. Adv. Integr. Anat. Evol. Biol..

[53] Snow M.H. (1978). An autoradiographic study of satellite cell differentiation into regenerating myotubes following transplantation of muscles in young rats. Cell Tissue Res..

[54] Liu Y., Chen S., Li W., Du H., Zhu W. (2012). Isolation and characterization of primary skeletal muscle satellite cells from rats. Toxicol. Mech. Methods.

[55] Seale P., Sabourin L.A., Girgis-Gabardo A., Mansouri A., Rudnicki M.A. (2000). Pax7 is required for the specification of myogenic satellite cells. Cell.

[56] Zhao Y., Cholewa J., Shang H., Yang Y., Xia Z. (2021). Advances in the Role of Leucine-Sensing in the Regulation of Protein Synthesis in Aging Skeletal Muscle. Front. Cell Dev. Biol..

[57] Cui C., Wu C., Wang J., Zheng X., Ma Z., Zhu P., Guan W., Zhang S., Chen F. (2022). Leucine supplementation during late gestation globally alters placental metabolism and nutrient transport via modulation of the PI3K/AKT/mTOR signaling pathway in sows. Food Funct..

[58] Liu T., Zuo B., Wang W., Wang S., Wang J. (2018). Dietary Supplementation of Leucine in Premating Diet Improves the Within-Litter Birth Weight Uniformity, Antioxidative Capability, and Immune Function of Primiparous SD Rats. Biomed Res. Int..

[59] Ko J.H., Olona A., Papathanassiu A.E., Buang N., Behmoaras J. (2020). BCAT1 affects mitochondrial metabolism independently of leucine transamination in activated human macrophages. J. Cell Sci..

[60] Waskiw-Ford M. (2020). Leucine-Enriched Essential Amino Acids Enhance Post-Exercise Muscle Recovery Independent Of ‘Free-Living’ Myofibrillar Protein Synthesis: 413 Board #229 May 27 9:30 AM–11:00 AM. Med. Sci. Sport. Exerc..

[61] Petrocelli J.J., Mahmassani Z.S., Fix D.K., Montgomery J.A., Reidy P.T., McKenzie A.I., de Hart N.M., Ferrara P.J., Kelley J.J., Eshima H. (2021). Metformin and leucine increase satellite cells and collagen remodeling during disuse and recovery in aged muscle. Fed. Am. Soc. Exp. Biol. J..

[62] Kakazu E., Ueno Y., Kondo Y., Fukushima K., Shiina M., Inoue J., Tamai K., Ninomiya M., Shimosegawa T. (2010). Branched chain amino acids enhance the maturation and function of myeloid dendritic cells ex vivo in patients with advanced cirrhosis. Hepatology.

[63] Master P.B.Z., Macedo R.C.O. (2020). Effects of dietary supplementation in sport and exercise: A review of evidence on milk proteins and amino acids. Nutrition.

[64] Chen X., Xiang L., Jia G., Liu G., Huang Z.J. (2019). Leucine regulates slow-twitch muscle fibers expression and mitochondrial function by Sirt1/AMPK signaling in porcine skeletal muscle satellite cells. Anim. Sci. J..

[65] She Y., Deng H., Cai H., Liu G. (2019). Regulation of the expression of key signalling molecules in mTOR pathway of skeletal muscle satellite cells in neonatal chicks: Effects of leucine and glycine–leucine peptide. J. Anim. Physiol. Anim. Nutr..

[66] Cirulli E.T., Singh A., Shianna K.V., Goldstein D.B. (2010). Screening the human exome: A comparison of whole genome and whole transcriptome sequencing. Genome Biol..

[67] Bigot A., Duddy W.J., Ouandaogo Z.G., Negroni E., Mariot V., Ghimbovschi S., Harmon B., Wielgosik A., Loiseau C., Devaney J. (2015). Age-associated methylation suppresses SPRY1, leading to a failure of re-quiescence and loss of the reserve stem cell pool in elderly muscle. Cell Rep..

[68] Edelman G.M. (1987). CAMs and Igs: Cell adhesion and the evolutionary origins of immunity. Immunol. Rev..

[69] Rowlands D.S., Nelson A.R., Raymond F., Metairon S., Mansourian R., Clarke J., Stellingwerff T., Phillips S.M. (2016). Protein-leucine ingestion activates a regenerative inflammo-myogenic transcriptome in skeletal muscle following intense endurance exercise. Physiol. Genom..

[70] Kwon O.S., Tanner R.E., Barrows K.M. (2015). MyD88 regulates physical inactivity-induced skeletal muscle inflammation, ceramide biosynthesis signaling, and glucose intolerance. Am. J. Physiol. Endocrinol. Metab..

[71] Tuttle C.S.L., Thang L.A.N., Maier A.B. (2020). Markers of inflammation and their association with muscle strength and mass: A systematic review and meta-analysis. Ageing Res. Rev..

[72] Alameddine H.S., Morgan J.E. (2016). Matrix Metalloproteinases and Tissue Inhibitor of Metalloproteinases in Inflammation and Fibrosis of Skeletal Muscles. J. Neuromuscul. Dis..

[73] Perandini L.A., Chimin P., Lutkemeyer D., Cmara N.O.S. (2018). Chronic inflammation in skeletal muscle impairs satellite cells function during regeneration: Can physical exercise restore the satellite cell niche?. Fed. Eur. Biochem. Soc. J..

[74] Beaudry A.G., Law M.L. (2022). Leucine Supplementation in Cancer Cachexia: Mechanisms and a Review of the Pre-Clinical Literature. Nutrients.

[75] Viana L.R., Canevarolo R., Luiz A.C.P., Soares R.F., Lubaczeuski C., Zeri A.C.d.M., Gomes-Marcondes M.C.C. (2016). Leucine-rich diet alters the 1H-NMR based metabolomic profile without changing the Walker-256 tumour mass in rats. BMC Cancer.

[76] Li Z., Zhang H. (2016). Reprogramming of glucose, fatty acid and amino acid metabolism for cancer progression. Cell. Mol. Life Sci..

[77] Kelly B., Pearce E.L. (2020). Amino Assets: How Amino Acids Support Immunity. Cell Metab..

[78] Nowosad A., Jeannot P., Callot C., Creff J., Perchey R.T., Joffre C., Codogno P., Manenti S., Besson A. (2021). Publisher Correction: p27 controls Ragulator and mTOR activity in amino acid-deprived cells to regulate the autophagy-lysosomal pathway and coordinate cell cycle and cell growth. Springer Sci. Bus. Media.

[79] Moro T., Ebert S.M., Adams C.M., Rasmussen B.B. (2016). Amino Acid Sensing in Skeletal Muscle. Trends Endocrinol. Metab..

[80] Sah N., Wu G., Bazer F.W. (2021). Regulation of Gene Expression by Amino Acids in Animal Cells. Amino Acids in Nutrition and Health.

[81] Kilberg M., Pan Y.-X., Chen H., Leung-Pineda V. (2005). Nutritional control of gene expression: How mammalian cells respond to amino acid limitation. Annu. Rev. Nutr..

[82] Hamanaka R.B., Nigdelioglu R., Meliton A.Y., Tian Y., Witt L.J., O’Leary E., Sun K.A., Woods P.S., Wu D., Ansbro B. (2018). Inhibition of phosphoglycerate dehydrogenase attenuates bleomycin-induced pulmonary fibrosis. Am. J. Respir. Cell Mol. Biol..

[83] Shen L., Hu P., Zhang Y., Ji Z., Shan X., Ni L., Ning N., Wang J., Tian H., Shui G. (2021). Serine metabolism antagonizes antiviral innate immunity by preventing ATP6V0d2-mediated YAP lysosomal degradation. Cell Metab..

[84] Kim S., Lee M., Song Y., Lee S.-Y., Choi I., Park I., Kim J., Kim J.-s., Seo H.R. (2021). Argininosuccinate synthase 1 suppresses tumor progression through activation of PERK/eIF2α/ATF4/CHOP axis in hepatocellular carcinoma. J. Exp. Clin. Cancer Res..

[85] Nilsson R., Jain M., Madhusudhan N., Sheppard N.G., Strittmatter L., Kampf C., Huang J., Asplund A., Mootha V.K. (2014). Metabolic enzyme expression highlights a key role for mthfd2 and the mitochondrial folate pathway in cancer. Nat. Commun..

[86] Kim D., Fiske B.P., Birsoy K., Freinkman E., Kami K., Possemato R.L., Chudnovsky Y., Pacold M.E., Chen W.W., Cantor J.R. (2015). SHMT2 drives glioma cell survival in ischaemia but imposes a dependence on glycine clearance. Nature.

[87] Martínez-Arnau F.M., Fonfría-Vivas R., Cauli O. (2019). Beneficial effects of leucine supplementation on criteria for sarcopenia: A systematic review. Nutrients.

[88] Lee S.Y., Lee H.J., Lim J.-Y. (2022). Effects of leucine-rich protein supplements in older adults with sarcopenia: A systematic review and meta-analysis of randomized controlled trials. Arch. Gerontol. Geriatr..

[89] Cruz B., Oliveira A., Gomes-Marcondes M.C.C. (2017). L-leucine dietary supplementation modulates muscle protein degradation and increases pro-inflammatory cytokines in tumour-bearing rats. Cytokine.

