# Supplementary material for: A Transcriptomic Regulatory Network among miRNAs, lncRNAs, circRNAs, and mRNAs Associated with L-leucine-induced Proliferation of Equine Satellite Cells
Source: Animals (Basel). 2023 Jan 6;13(2):208. doi: 10.3390/ani13020208 (PMC9854542; doi:10.3390/ani13020208)
Supplement: Supplementary file 1 [file animals-13-00208-s001.zip › Table S1.pdf]

Table S1 Primers used for quantitative Real- Time PCR

| Gene Symbol | Forward primer | Reverse primer | Annealing temperature (℃) | Product length (bp) |
|---|---|---|---|---|
| GXYLT2 | TTTGCTCGGCACCCTTTCTAT | TGATGGCATTCTTGTACTTCTGG | 60 | 166 |
| EPHA2 | CAGTTCAGCCACCACAACATCAT | GCTACCGACAGAGGCCCTATTC | 60 | 161 |
| ANKRD2 | GCAGACAGCAATGTGAGGGATAA | GTCCAGGCCCAGGGATAGAA | 60 | 105 |
| MATN2 | AGAGGAAGTCTGAAGTGGAGCGT | GCCTCCCATCTGTCACAATCA | 60 | 174 |
| AEBP1 | GCCACCCAGTGCAACTTCAT | ACGGAGCCGCATCTGTTCA | 60 | 186 |
| CPT1A | GCTGGGTTATGCAGAGGATGG | GTGGAACGGGAAAGAATGGA | 60 | 172 |
| HSD11B2 | GCTGTGACTCTGGTTTTGGCAA | GGCACCAGGGCTATCCAAT | 60 | 98 |
| TNNT3 | ACTCACTGCTCCTAAGATCCCG | GCAGCTCCATGAGGTCTTTGTTC | 60 | 89 |
| GSN | ACCTGGGCAACAACATCTATCA | CCTTCCTCAAACACGGACACT | 60 | 136 |
| ADM | GGAACTTCAGGTGTCCAGCA | GAAGTTGTTCATACTCTGGCGAT | 60 | 172 |
| GAPDH | GCCAAATACGATGAGATCAAGAAG | CATACCAGGAAATGAGCTTGACAAA | 60 | 187 |
