# Supplementary figures and images for: A Transcriptomic Regulatory Network among miRNAs, lncRNAs, circRNAs, and mRNAs Associated with L-leucine-induced Proliferation of Equine Satellite Cells
Source: Animals (Basel). 2023 Jan 6;13(2):208. doi: 10.3390/ani13020208 (PMC9854542; doi:10.3390/ani13020208)

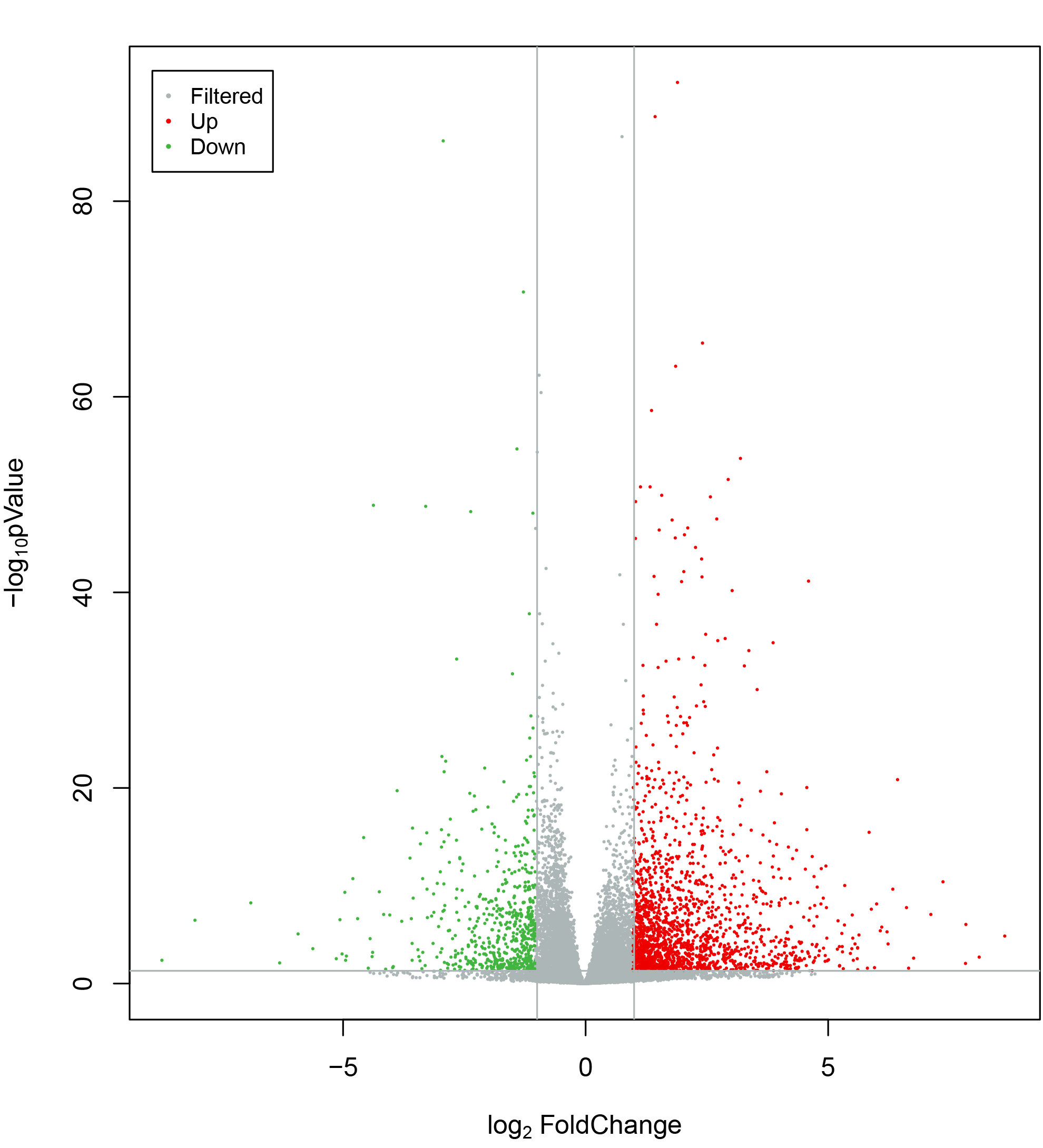

Supplement: Supplementary file 1 [file animals-13-00208-s001.zip › Figure S1.tif]
